# The Bidirectional Relationship between Periodontal Disease and Diabetes Mellitus—A Review

**DOI:** 10.3390/diagnostics13040681

**Published:** 2023-02-11

**Authors:** Ioana Păunică, Marina Giurgiu, Anca Silvia Dumitriu, Stana Păunică, Anca Mihaela Pantea Stoian, Maria-Alexandra Martu, Cristian Serafinceanu

**Affiliations:** 1 Department of Diabetes, Nutrition and Metabolic Diseases, Carol Davila University of Medicine and Pharmacy, 8, Eroii Sanitari Boulevard, 050471 Bucharest, Romania; 2 Department of Periodontology, Carol Davila University of Medicine and Pharmacy, 17–23, Calea Plevnei, 010221 Bucharest, Romania; 3 Department of Periodontology, Grigore T. Popa University of Medicine and Pharmacy, Universitatii Street 16, 700115 Iasi, Romania

**Keywords:** diabetes mellitus, periodontitis, inflammation, microvascular complications, oral microbiota, tobacco

## Abstract

Periodontitis is a chronic inflammatory disease caused by the presence of a bacterial biofilm known as dental plaque. This biofilm affects the supporting apparatus of the teeth, especially the periodontal ligaments and the bone surrounding the teeth. Periodontal disease and diabetes seem to be interrelated and in a bidirectional relationship, and have been increasingly studied in recent decades. For example, diabetes mellitus has a detrimental effect on periodontal disease, increasing its prevalence, extent, and severity. In turn, periodontitis negatively affects glycemic control and the course of diabetes. This review aims to present the most recently discovered factors that contribute to the pathogenesis, therapy, and prophylaxis of these two diseases. Specifically, the article focuses on microvascular complications, oral microbiota, pro- and anti-inflammatory factors in diabetes, and periodontal disease. As presented in this review, these two diseases require specific/ complementary therapeutic solutions when they occur in association, with new clinical trials and epidemiological research being necessary for better control of this interdependent pathogenic topic.

## 1. Introduction

Periodontal disease has been linked to a multitude of systemic diseases and conditions including cardiovascular, renal, autoimmune, pulmonary, endocrine and neurodegenerative diseases and cancer, to name a few [1,2,3,4,5,6,7]. To what degree this relationship is causal is a matter of debate. In order to better grasp the connections that link various conditions, future studies will have to decipher the complex networks that actuate the biological processes behind the conversion of physiological to pathologicalentities.

The association between periodontal disease (PD) and diabetes is relatively commonly described in the literature. This is caused by multiple and complex mechanisms, being determined both by common etiopathogenic factors and by overlapping phenomena due to high prevalence characteristics [8].

Several studies show that patients with either neglected diabetes or having difficulty controlling their serum glucose level have a 2–3 times higher risk of developing periodontitis (the level of glycemic control being the critical determining risk factor) [9]. In addition, long-term studies have shown a higher incidence of progressive periodontitis in patients with diabetes. For example, cross-sectional epidemiologic studies have demonstrated that patients with periodontitis suffer a more extensive and severe loss of periodontal tissue support when diabetes is associated [10]. Numerous controlled human studies demonstrate that successful periodontal treatment reduces circulating C-reactive protein (CRP) and tumor necrosis factor (TNF)-α levels in subjects with diabetes, proving its active role in inflammation [11]. 

As suggested by data from the literature, the interrelation between periodontal disease and diabetes mellitus appears to be bidirectional. Thus, periodontal disease is currently considered the sixth complication of diabetes; periodontitis affects the prevalence, evolution and therapeutic management of diabetes [12,13]. Periodontal disease can have a significant impact on a diabetic’s metabolic state. According to recent literature data, periodontal disease treatment may help to improve glucose control [14,15]. The increased levels of pro-inflammatory factors in the gingiva of patients with poorly controlled diabetes suggest the existence of a biological pathway that may aggravate periodontitis [11].

Diabetes and periodontal diseases can also coexist, both diseases being highly prevalent in modern societies. Thus, the overall prevalence of diabetes was estimated at 9.3% (463 million people) in 2019 [16] and is expected to increase [17]. Regarding periodontal disease, there is a prevalence ranging from 20% to 50% worldwide; however these percentages are expected to rise [18]. This is partly a consequence of the ageing population, nonetheless, a significant reduction in tooth loss in the elderly is also associated with various oral pathologies [18].

From an age-related point of view, most diabetics are between 45 and 64 years old in developing countries and about 65 years old or older in developed countries [19]. This pattern will probably be accentuated by the year 2025 due to a higher concentration of subjects in urban areas, as already observed in current studies [12,16]. In 2007, diabetes affected approximately 250 million people worldwide, a figure which will reach 350 million by 2030 [20]. Similarly, the global elderly population increased from 382 million in 1980 to 962 million in 2017 and is expected to reach 1.4 billion by 2050. The global prevalence of periodontal disease is likely to also increase in the coming years due to growth in the ageing population and increased retention of natural teeth due to a significant reduction in tooth loss in the older population [18]. Thus, periodontal disease and diabetes are common diseases in modern societies and have negative impacts on health and quality of life [21]. 

Starting from these observations, the aim of this narrative review is to present recent data on periodontitis and diabetic diseases in light of new discoveries in areas such as microbiome studies, comorbidities, cytokine pathways and common genetic implications. Furthermore, practitioners from both fields will benefit from the presented new data in order to better understand the bidirectional interrelations between these distinct pathological entities and provide comprehensive and quality care for patients. Finally, our purpose is to establish and provoke debate about how these two diseases can be best prevented but also treated as effectively as possible when they are associated. 

To assess the interaction between diabetes mellitus and periodontitis, we carried out a search of the following electronic databases: Web of Science, PubMed and Scopus. To do so, we used the following terms: ‘periodont *’, ‘diabetes’, ‘pre-diabetes’, ‘hyperglycemia’, ‘microbiome’, ‘cytokines’, ‘gene polymorphisms’. Only pertinent literature in English published between January 2013 and December 2022 was considered for this search. Particular attention was given to notable journals, systematic reviews and meta-analyses. Two reviewers independently assessed potential abstracts as well as full texts, and agreement was reached through discussion regarding the papers considered suitable for inclusion in this review.

## 2. Etiopathogenesis and Clinical Correlations between Diabetes Mellitus and Periodontal Disease

Numerous cross-sectional, case–control, cohort and interventional studies have been used to extensively study the association between periodontal disease and diabetes. It has been the topic of many scientific literature reviews [8,9,10,11,12,14,15,17,19,22]. In 1990, Nelson et al. studied the incidence and prevalence of periodontal disease and its relationship to the non-insulin-dependent diabetes mellitus of Pima Indians [23]. This population presented a high prevalence of T2DM (type 2 diabetes). In the 3219 subjects analyzed, the prevalence of periodontitis was statistically higher in patients with T2DM than in normal subjects [23].

A study in Saudi Arabia investigated a possible bidirectional relation between periodontitis and type 2 diabetes. Quadri et al. designed a 2:1 sample for control patients, with a mean age of study sample of approximately 38 years old. The results showed a significant association between T2DM and hypertension with periodontitis; moreover, the study showed that cigarette smoking, use of khat (widespread in this specific area), and poor oral hygiene were significantly associated with periodontal disease [14].

Another study, conducted at the Altamash Institute of Dentistry/Pakistan on 129 patients, divided subjects into four groups depending on whether or not they had diabetes. After this, periodontal disease patients were followed up at 3 and 6 months. Non-surgical and surgical treatment techniques for periodontal disease have been applied for pocket probing depth (PPD) 4 < 6 mm, non-surgical treatment options included root planning, as well as supragingival and subgingival scaling. Modified flap surgery with scaling and root planning eliminated PPD ≥ 6 mm [24]. After three months of treatment, people with diabetes (whether or not they have PD) had a mean reduction in HbA1c levels of 0.3%, while at six months, the average decrease in HbA1c levels was 1% and 0.8%, respectively [25]. In addition, there was only a slight improvement in periodontal status in diabetic and non-diabetic subjects with PD compared to six-month follow-ups. The overall conclusion of this study was that, in each of the three check-ups, patients with diabetes and PD or non-diabetics with PD had more significantly increased PPD than diabetics without PD or non-diabetics without PD [24]. 

Several studies found a moderate reduction of 0.4% in the mean HbA1c value among diabetic patients treated non-surgically for PD [24,25]. After three months, the mean decrease in HbA1c levels in diabetic subjects receiving scaling was 0.3%. A 1% reduction in HbA1c levels resulted in a 37% reduction in microvascular complications and a 21% reduction in diabetes-related deaths [24]. 

One study that analyzed whether surgical oral interventions such as dental extractions have an influence on certain molecules (hepatocyte growth factor (HGF), tumor necrosis factor-α (TNF-α), interleukin 18 (IL-18), matrix metalloproteinase 9 (MMP-9)) and bone destruction markers such as osteoprotegerin (OPG), kappa B nuclear factor receptor activator ligand (RANKL) and oxidative stress markers—total oxidant status (TOS), total antioxidant capacity (TAC), and whether there is a correlation with HbA1c levels. T2DM subjects presented elevated TNF-α, IL-18, MMP-9, TOS, OPG, and RANKL when compared to both pre- and post-extraction controls. At baseline IL-18, TNF-α, MMP-9, OPG, RANKL and TOS were good predictors of HbA1c levels, while 3 months after the surgical intervention, there was a significant correlation between oxidative status biomarkers and HbA1 [26].

To assist in understanding these etiopathogenetic and clinical correlations between diabetes mellitus and periodontal disease, specific data related to the onset and evolution of the two diseases are presented below.

## 3. Diabetes Mellitus

Diabetes mellitus is a complex disease that includes several metabolic dysfunctions caused by a long-term state of hyperglycemia. The hyperglycemic status is generally a consequence of decreased insulin secretion and action. Diabetes mellitus can have an insidious/silent onset, with a family history often being described [27,28].

Early symptoms include several manifestations (thirst, polyuria, polydipsia, polyphagia, blurring vision, and weight loss); the intensity and association depend on the time of diabetes diagnosis. In advanced stages, chronic hyperglycemia modifies the structure and function of various organs, such as the eyes (retinopathy), kidney (nephropathy), heart (cardiovascular diseases), brain (cerebrovascular diseases, stroke, cognitive dysfunction), nerves (neuropathy) blood vessels (increased risk of the atherosclerotic process) and increases the risk of infections. As a result, the complications of uncontrolled diabetes are not only severe but also life-threatening. Furthermore, the increased risk of death, for example, heart disease or stroke, is often two to four times more common in such cases than in normal subjects [29,30,31].

**Figure 1:**
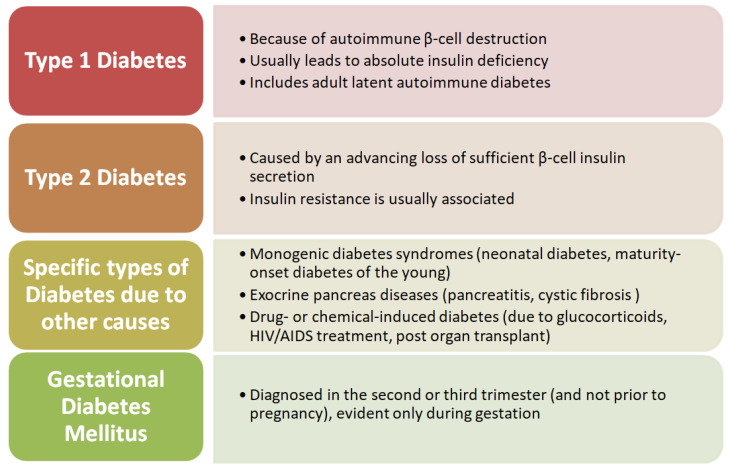
Schematic representation of the classification of diabetes.

Classically, diabetes mellitus is classified into autoimmune (T1DM) and non-autoimmune forms (T2DM). From a clinico-pathological perspective, other subtypes of diabetes have been described, such as monogenic diabetes (maturity-onset diabetes of the young or neonatal diabetes), gestational diabetes, and a possibly late-onset autoimmune form (latent autoimmune diabetes in the adult) (Figure 1) [32,33,34,35].

Type 1 diabetes is a chronic illness, characterized by the body’s inability to produce insulin due to the autoimmune destruction of beta cells in the pancreas. It is the most common type of diabetes in children. Although the disease’s onset is most frequently seen in children, it can also manifest in adults [35,36].

Type 2 diabetes mellitus (T2DM) is characterized by insulin resistance, inflammation, advanced glycation end product accumulation, and increased oxidative stress. It is usually associated with overweight/obesity and increased abdominal circumference [37,38,39].

Regardless of the type, diabetes is associated in the long term with changes in the large and small blood vessels (macrovascular and microvascular alterations). Diabetes also appears to modify the proprieties of macrophage cells, decreasing the response to bacterial infections and wound healing. In a hyperglycemic status, oxidation and glycation of proteins and lipids accumulate advanced glycation end products (AGEs). The AGE receptors (RAGE) are generally found on endothelial and inflammatory cells [20,31,40]. 

## 4. Microvascular Complications

As presented above, chronic exposure to hyperglycemia leads to microvascular injury in diabetic patients. The intensity of these lesions depends on the time from diabetes onset, the association of increased blood pressure and cholesterol, and genetic predisposition [30]. Diabetic microvascular lesions mainly affect the kidneys, the eyes, and the nervous system. Diabetic nephropathy and retinopathy are described in 25% of patients with T2DM cases, whereas diabetic neuropathy is identified in about 50% of diabetic patients. Other tissues/ organs are also affected, such as the oral cavity (periodontal disease), genital organs (erectile dysfunction affecting 35–90% of diabetic men), muscle, skin (diabetic dermopathy), heart (diabetic cardiomyopathy), etc. [30,41,42,43]. 

Studies describe pathological changes in the gingival vascularization of patients and animals with diabetes. Such changes include basement membrane thickening, angiogenesis, and increased osmotic tissue pressure. Therefore, diabetes affects the periodontal tissue through vascular lesions (similar to neural, retinal, renal, and other affected tissues), caused by multiple mechanisms, as described below [44,45,46].

Several factors contribute to microangiopathy in diabetes: a shortage of nitric oxide (NO), oxidative stress and inflammatory factors. Increased reactive oxygen species (ROS) levels in diabetes are attributed to increased ROS generation and impaired antioxidant defense (resulting from mitochondrial collapse) [47,48]. Endothelin-1 is also involved in the pathology of diabetes, as studies on mice proved that peptide endothelin ET-1 traps have a significant therapeutic effect. The serum level of vascular endothelial growth factor (VEGF) is often higher in people with diabetes, leading to increased angiogenesis. However, the expression of VEGF is variable, being increased significantly in the kidney and retina of diabetes patients [49,50].

These factors disrupt the endothelial barriers and up-regulate integrin expression, leading to vascular leakage and abnormal permeability [51,52]. Endothelial dysfunction in the microvascular area is relatively difficult to measure. Instead, it is estimated by measuring eNOS and the expression of the adhesion molecule, the extent of penetration of white blood cells, changes in capillary density, and finally, the appearance of lesions [50].

A multicenter, cross-sectional study conducted in Japan included 620 type 2 diabetes patients. The prevalence and severity of periodontitis were compared between patients with ≥1 microvascular complication (neuropathy/retinopathy/nephropathy) and those with none. The results showed greater severity of periodontal disease in patients with more than one complication. Additionally, patients with three microvascular complications had a more severe case of periodontitis than those with one [53]. 

## 5. Periodontal Disease

Periodontal disease is generally a chronic condition caused by a long-lasting inflammatory process in the supporting tissues of the teeth (gingiva, periodontal ligament, alveolar bone). The chronic inflammatory process induces progressive destruction of these periodontal tissues, which leads to the progressive loss of tooth attachment (CAL) [54]. The most common etiopathogenic factors are poor oral hygiene (creating favorable conditions for developing dental bacterial biofilm), smoking, and a predisposition to chronic inflammation [15,55,56]. 

The incidence of periodontitis appears to depend on additional favorable factors, such as social status and age. Thus, low-income people are 1.8 times more likely to develop severe periodontitis than those with high incomes [8,18,57]. The onset of chronic periodontitis is correlated with certain age groups, as the severity increases with age. In an epidemiological study, the highest prevalence was found in the elderly population (82%), followed by adults (73%) and adolescents (18.8%) [18].

Periodontitis is a condition with progressive evolution, being reversible in the early stages when gingivitis regresses through proper oral hygiene. Unfortunately, the initial signs are faint, delaying the diagnosis and treatment from onset [58]. In advanced stages, inflammation and tissue degradation become irreversible, leading to loss of alveolar bone, tooth mobility, and even tooth loss [58]. Consequently, patients with progressive periodontal disease often have mastication and food intake problems, negatively affecting their nutritional and general health [49,59,60].

**Figure 2:**
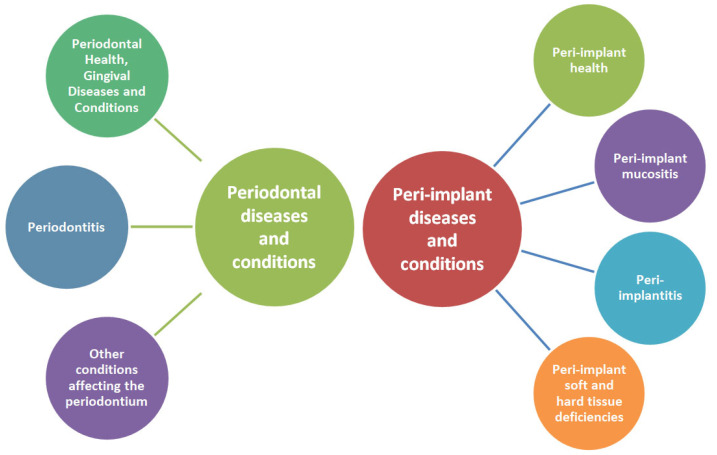
Schematic representation of the classification of periodontal and peri-implant diseases and conditions.

Since 2017, a new classification of periodontal and peri-implant diseases has been introduced that better encompasses the complexity of periodontal conditions and iss in accordance to new medical discoveries. It will also support dental professionals in prevention and elaborating a correct diagnosis and treatment plan. Figure 2 is a simplified illustration of this classification [61]. 

The clinical diagnosis is established based on the manifestations described above. Periodontal pockets form when collagen fibers in the periodontal ligament are damaged. Therefore, septic systemic manifestations may also be present due to harmful endotoxins and exotoxins that enter the bloodstream (by epithelium destruction of the periodontal pocket) [49]. Investigative clinical indexes used for diagnostic purposes consist of bleeding on probing (BOP), pocket probing depth (PPD), clinical attachment level (CAL), and radiological assessment, all being widely used and documented. 

The treatment of periodontitis requires professional care, consisting of root surface debridement. Medical care must be associated with proper oral hygiene, which aims to reduce the detrimental effects of dental bacterial biofilm [55,62]. This involves both educating and motivating the patient to optimize oral hygiene and decrease or eliminate risk factors such as smoking [63].

## 6. Oral Microbiota

The microbiome and the human body are in a complex biological interrelation in symbiosis, with considerable benefits. This symbiosis results from millions of years of co-evolution, mutual adaptation, and functional integration, leading to a diverse oral microbiome [11,64]. 

Such biofilm formation can occur in several places of the oral cavity: in cavities within teeth, near the dental pulp (fissure biofilm), on the dental enamel adjacent to the gingiva (supragingival biofilm), in the subgingival area (primarily composed of anaerobe species) and on artificial surfaces (e.g., dental fillings) [65,66]. 

Dental plaque is formed in an ordered sequence of events, resulting in a structurally and functionally organized microbial community with high species diversity. Distinct stages of plaque formation include acquired pellicle formation; reversible adhesion, involving weak long-range physicochemical interactions between the cell surface and the pellicle, which can result in stronger adhesin receptor-mediated attachment; co-adhesion, which results in secondary colonizers to adhere to pre-attached cells, multiplication and biofilm formation, and, finally, detachment [64,67,68]. 

The balance between the human microbiome and the host is essential for maintaining the health of the body. Breaking this balance leads to dysbiosis and is described in several systemic disorders, such as asthma, atopic diseases (inflammatory or autoimmune diseases), obesity, hypertension, depression, cognitive disorder, vascular/neuronal diseases, metaplasia, etc. Dysbiosis can be both a contributing factor and the effect of the systemic diseases presented, thus having an essential role in the occurrence and evolution of several conditions [69,70,71,72].

In a recent study, five hundred participants from three different nursing homes in Japan participated in a study investigating the salivary microbiota. The analysis of the microbiome of the saliva samples identified over 700 bacterial species. Genomic DNA extraction from 15 elderly patients (3 of them with T2DM) showed that the oral bacterial population in the T2DM group was different from that corresponding to the non-diabetic group [73].

A study conducted in South Africa on 128 patients examined the bacterial composition in plaque samples. The results identified changes in the design of the oral microbiota in the presence of altered glycemic status and different stages of periodontal disease. However, whether the differences obtained are the consequence or cause of periodontal disease or hyperglycemia is still uncertain [74].

*P. gingivalis* is a major periodontopathogen that has also been linked to. A study on 37 subjects with diabetes with or without periodontitis observed that *P. gingivalis* was detected in 27.03% percent of analyzed patients [75]. Similar results were observed by Aoyama et al., who also noted aggravated CAL (clinical attachment level) and BOP (bleeding on probing) in uncontrolled DM patients [76].

Some have hypothesized that gut microbiota could provide a link between diabetes and periodontal disease. Following ligature-induced periodontitis, gut microbiota of mice was analyzed through gene sequencing. The researchers observed that in the periodontitis group several bacteria that produce butyrate bacteria were decreased; furthermore, higher levels of fasting blood glucose, HbA1c, and glucose intolerance were noticed. Moreover, fasting blood glucose decreased significantly when periodontitis was eliminated [77]

Contrary to the results of these studies, Castrillon et al. highlight the fact that diabetic subjects had lower levels of *P. gingivalis, T. forsythia* and *T. denticola*, but higher counts *A. actinomycetemcomitans*, vs non-diabetics (*p* < 0.05). *P. gingivalis* was linked to periodontal disease only in systemically health patients, while *A. actinomycetemcomitans* was associated with periodontitis in subjects with diabetes [78].

There is an indication that variations in the microbial profile of diabetics and non-diabetics exist. *T. forthysia* is reportedly lower in levels in subjects with DM and periodontitis, while less agreement is found regarding *P. gingivalis* and *A. actinomycetemcomitans* [79]. A study that compared periodontal status and *P. gingivalis* and *P. intermedia* counts in pregnant women with and without gestational diabetic mellitus highlighted that diabetics had elevated periodontal disease index scores, gingival index and probing depth vs non-diabetics, concluding that there is a significant association between periodontal disease severity and elevated levels of *P. gingivalis* and *P. intermedia* in gestational diabetic women [80].

Even though some microbial species might have increased counts in patients with diabetes and periodontitis, respectively, the numbers might decrease in patients with both pathologies co-existing. This may be due to the shift in genera abundance caused by the additional stress generated by both diseases co-existing [81,82].

Differences in the subgingival microbiome were assessed with metagenomic sequencing analysis in subjects with periodontitis and/or diabetes mellitus type 2, and modifications of the periodontal sulcus microbiology were less prominent in diabetes patients in healthy versus periodontitis oral status. In periodontitis, glucuronate and pentose interconversion, mannose, galactose and fructose metabolism were up-regulated, while in diabetes sulfur metabolism, bacterial secretion system, glycolysis pathways and lipopolysaccharide, and phosphotransferase and peptidoglycan biosynthesis were heightened. These pathways could constitute potential bacterial functional associations between diabetes and periodontal disease [83]. 

Another study that found similar results obtain from using metagenomic shotgun sequencing to perform a longitudinal analysis of the subgingival microbiome in type 2 DM subjects versus nondiabetics in relation to periodontal status. Patients were into divided healthy, periodontitis and resolved states after treatment. The conversion of the subgingival microbiome from healthy to periodontitis was less important in diabetic subjects versus non-diabetics, even though oral clinical status was similar between the two. Moreover, in diabetics there was an abundance of pathogenic species. This was not only the case in periodontitis-affected subjects, but also in healthy ones, implying a heightened risk of progression to periodontal disease [84].

When considering periodontitis patients with diabetes compared to periodontitis systemically healthy, ones a lower concentration of Streptococcaceae, Veillonellaceae and Pasteurellaceae and higher abundance of Neisseriaceae and Leptotrichiaceae was noticed, while Porphyromonadaceae was increased in both [85]. Microenvironments which are high in glucose may dictate the filtering of the habitat to favor glucose- and protein-rich, anaerobic, pro-oxidant, thriving bacteria. This is supported by the fact that hyperglycemic subjects have high counts of Propionibacterium, Corynebacterium, Sphingomonas, Capnocytophaga, Neisseria, Pseudomonas and Bergeyella [86]. 

An animal study not only highlighted that diabetes causes modifications to the oral microbiome, but it also illustrated how the transposition of modified microbiota in germ-free mice leads to heightened periodontal inflammation and bone loss in comparison to transference of microbes from mice with normal glycaemia. Anti-interleukin-17 administration modified the bacterial composition and led to diminished bone loss in germ-free mice versus germ-free mice that received oral bacteria from control diabetic mice [87]. 

In these circumstances, we can assume that certain subjects could be more prone to dysbiotic modifications of the subgingival microbiome, possibly because of affected immune system pathways and metabolic regulation that can encourage inflammophilic species multiplication. 

## 7. Smoking as a Predisposing Factor for Periodontal Disease and Diabetes

Routine dental care involves a complete medical history of all patients from the first presentation. For diabetic patients, it is essential to determine the specific treatment and if the level of glycemia is controlled [9]. Periodontal disease is generally multifactorial, so effective disease management involves investigating and identifying all relevant risk factors. Periodontitis is related to age, poor oral hygiene, smoking, obesity, socioeconomic status, and chronic conditions like cardiovascular disease, osteoporosis, and diabetes [88,89]. The most common risk factors for periodontal disease are pathogenic bacteria, calculus, smoking and diabetes [90]. Regardless of the consumption model, tobacco is associated with an increased risk of developing severe forms of periodontal disease [68]. In diabetic patients, periodontal disease is more severe in smokers than in non-smokers. Both active and passive smokers have an increased risk of developing micro-and macrovascular complications [90]. 

The mechanisms by which tobacco increases the risk of periodontitis are reduced gingival perfusion (which decreases the transport of nutrients, oxygen, and elimination of final metabolic products), suppression of the immune response (especially inflammation), suppression of morphological and functional regeneration of periodontium, dysbiosis and thus increased infectivity of the oral microbiota [90]. The mechanism of smoking that leads to vasoconstriction is the reduction of endothelial nitric oxide (NO) synthesis. It is caused by suppression of endothelial NO synthase (eNOS) expression in the vascular wall and decreased NO-mediated oxidative stress [91]. The gaseous component of tobacco smoke contains many reactive oxygen species (ROS) which are generated during combustion. Such compounds act on the endothelium, increasing the production of lipid peroxides that destroy NO and inhibit eNOS, thereby decreasing NO’s bioavailability. The reduced NO level increases the vascular tone, leading to vasoconstriction and increased blood pressure [91].

A study conducted in Brazil on 102 patients with periodontal disease evaluated the effects of type 2 diabetes mellitus and smoking on pro-and anti-inflammatory cytokines. Patients were included in a cross-sectional study and were assigned to four groups: non-smokers and non-diabetics; diabetes only; smoker only; both smokers and diabetics. Levels of 13 pro-inflammatory (IFN-γ, TNF-α, MIP-1α, GM-CSF, Interleukin (IL)-1β, IL-2, IL-6, IL-7, IL-8, IL-12, IL-17, IL-21, IL-23) and 5 anti-inflammatory cytokines (IL-4, IL-5, IL-10, IL-13, and TGF-β) were evaluated. The results obtained showed that the ratio of pro-and anti-inflammatory cytokines varied by group [92]. The result concluded that diabetes mellitus induced a general pro-inflammatory state, while smoking promoted immunosuppression in periodontal tissues affected by periodontitis. Therefore, even though diabetes and smoking are recognized as risk factors for periodontal disease, their mechanisms of action are distinct. In addition, in cases where the two risk factors are associated, smoking seems to promote the hyperinflammatory effect of diabetes [92].

Another study that analyzed the microbiome of 175 subjects divided them into various groups in order to evaluate the biodiversity of periodontal pocket microbes and their co-occurrence patterns. These groups are: non-diabetic non-smoking subjects (control group), smokers, diabetics, diabetic smokers with periodontitis, and periodontally healthy controls (smokers and diabetics). When both diabetes and smoking coexist in the same subjects, their collective effect in terms of the impact on the subgingival microbiome surpasses each influence considered separately. As such, it is important to implement prompt intervention strategies in order to preserve healthy oral cavity ecology [93]. 

## 8. Pro and Anti-inflammatory Factors

Several factors mediate chronic inflammation. For example, proinflammatory cytokines (IL-1β, TNF-α, IL-6, and IL-17) lead to periodontal tissue inflammation, which induces local lesions. IL-1ß is a highly potent proinflammatory mediator that causes vasodilatation prostaglandin expression and promotes the attraction of granulocytes to tissue. Therefore, cytokine-based therapies have improved periodontitis and overall health [94]. In addition to the immune system, other functions that do not directly involve the immune system also intervene, such as the activation of osteoclasts with bone resorption [45,95]. 

Another factor that should be considered is the gingival fluid, generated by a physiological process, that implies a continuous flow of serum is transudated to the gingival sulcus. In gingivitis, the transuded gingival fluid becomes an exudate, which contains increased microbial and host-derived substances. Such an exudate involves pro-inflammatory cytokines (IL-1ß, IL-6, PG-E2, TNF-α) and immune cells or enzymes released by recruited and resident cells, all this being the expression of local inflammatory reaction [60]. The composition of the exudate appears to depend on the mediator studied. In patients with T_2_DM and chronic periodontitis, some studies showed an increased level of IL-1ß in the gingival crevicular fluid. At the same time, other published data did not report statistically significant differences compared to people with only one of the two diseases [94,96].

Regarding diabetes, it has been reported that inflammatory factors such as TNF-α, IL-1ß, IL-6, and IL-18 are increased in diabetic patients, contributing to insulin resistance through the JNK and IKKb/ NFkB pathways [4]. IL-1β also induces pancreatic β-cell apoptosis (related to the pathogenesis of T2DM) and promotes intimal inflammation and atherogenesis [45,95]. Increased production of inflammatory cytokines thus contributes to insulin resistance and the destruction of pancreatic beta cells, which significantly facilitates the onset of diabetes complications [74]. Accordingly, inflammatory mediators play a dual role in T2DM and contribute to hyperglycemia-induced insulin resistance and diabetic complications [97,98]. Adipose tissue appears to be an important site for producing inflammatory mediators due to crosstalk between adipocytes, macrophages, and other immune cells that infiltrate expanding adipose tissue. Consequently, it contributes to the insulin resistance induced by hyperglycemia and thus leads to diabetic complications [74]. 

Data on anti-inflammatory proteins are relatively limited, as it is currently supposed that the imbalance between pro- and anti-inflammatory cytokines is essential for developing diabetes mellitus [99,100]. In support of this perspective, several studies have shown that IL-1ß receptor antagonists improve glycemic control and counteract the destruction of ß-cells [101]. Adiponectin, a hormone with anti-inflammatory and insulin-sensitizing effects, is associated with a decreased risk of T2DM. On the other hand, patients with T1DM have higher circulating levels of adiponectin, which could be explained by their autoimmune status [100].

Taylor et al. presented in 2013 that there is good evidence of increased IL-1β, IL6, and RANKL/OPG activity in patients with both diabetes and periodontitis (compared to patients with only periodontal disease) and that there is a significant relationship between the activity of these cytokines and glycemic control [102].

## 9. Common Genes and Pathways in Periodontal Disease and Diabetes

Diabetes mellitus and periodontal disease are both considered amalgamate types of diseases. Although they have different determining factors, they share many common environmental and genetic factors that trigger and regulate the diseases. Thus subjects respond distinctively to shared environmental demands, this variation in response is in part attributed to the genetic profile. Nowadays it is certain that genes play a major part in the predisposition and advancement of both pathologies. Proclivity and disease severity are a result of the synergic interaction between polymorphisms and genetic mutations and the various environmental agents [103].

As previously discussed, diabetes and periodontitis have several cytokinic pathways in common. A study assessed glucose levels, clinical (pocket probing depth (PPD), gingival recession (GR), clinical attachment level (CAL), and bleeding on probing (BOP)) and immunological parameters in T2DM subjects vs systemically healthy controls. Twenty-one patients underwent tooth extraction surgery and gingival sample was collected for mRNA, cDNA analysis to evaluate gene expression of TNF-α, NF-kB, IL-1-β, IL-6 and IL-10. The clinical (BOP) and glucose parameters (fasting glucose levels, and HbA1c) were statistically higher in the diabetes group; moreover, RNAm levels of IL-1β, TNF-α, and NF-kB were higher when compared to healthy controls. Thus the authors concluded that diabetes and hyperglycemic status elevate pro-inflammatory immune factors levels but also severity of periodontitis [104]. On the other hand, a meta-analysis that evaluated data concerning the correlation between periodontal disease and genetic polymorphisms in interleukins in subjects with and without diabetes did not find any definite link [105].

Other studies have pointed out that polymorphisms of MMP-8 -381A/G (rs1320632) and -799C/T (rs11225395) are potential risk factors for several pathologies such as cancer, carotid atherosclerosis, HIV, periodontal disease and diabetes [106,107,108,109,110]. Furthermore, elevated MMP-8 levels in serum are also connected to TNF-α expression, and related to an exacerbated inflammatory status [111].

Considering the fact that polymorphisms in the KCNJ11, HNF1A, IRS1, TCF7L2, CDKAL1, CDKN2B, RPSAP52, GPR45 HHEX, IL18, and RUNX2 genes have been connected to type 2 diabetes mellitus (T2DM) and/or periodontitis by genome-wide association studies there is a strong incentive for further studied on this matter [112]. 

An interesting study assessed expression of immune-related genes in dyslipidemia, type 2 diabetes mellitus and periodontitis individuals. IL10 and IFNA genes were elevated in dyslipidemic subjects while STAT3, IRF1, JAK1, IFNG and IP10 were diminished. IL10 genes correlated positively with periodontal, glucose and lipid profiles, whereas IFNG (pro-inflammatory) correlated negatively [113].

Similarly, lipid metabolism gene polymorphisms were linked to an increased likelihood for T2DM-periodontitis comorbidities while also proving gene-sex interaction. A relevant gene marker appears to be APOB-rs1042031, shown to be linked to obesity, lipid metabolism and glucose profiles, as well as with periodontal disease [114].

Even though a myriad of cytokines participating in inflammation could be proposed as crucial factors in both pathologies, they could simply be factors that modify and regulate the pathogenesis of the diseases; as such, experimental or clinical studies have limited value in clearly displaying the shared pathogenic pathways. This being said, microarray analysis through the integration and analysis of data can entail new and decisive information about the pathological process. A study that utilized this technique sought to determine possible biomarkers of diabetes and periodontitis via bioinformatics methods through the integration of expression data from several independent cohorts. The researchers established 152 commonly differentially expressed genes (27 downregulated and 125 up-regulated genes) and revealed three highly connected hub genes that were up-regulated in both diabetes and periodontitis and were especially enriched in the Fc gamma R-mediated phagocytosis pathway, and could represent promising therapeutic targets in subjects with both diseases [115].

A case–control study investigated gene-environment interplay amid moderate or severe periodontitis, adiponectin (ADIPOQ)-rs1501299 and leptin receptor (LEPR)-rs1137100 polymorphisms on type 2 diabetes and found an increased risk even after adjustment for smoking status, gender, age, economic status, BMI, hypertension and alcohol consumption [116].

*P. gingivalis* could provide insight on how the host interactome is modified in cognitive disorders, cardiovascular disease, diabetes mellitus and obesity. Genome-wide association studies indicated that the host genes of the *P. gingivalis* interactome were significantly enriched in these pathologies, suggesting significant gene/environment interplay between this periodontal pathogen and gene expression modifications or susceptibility genes in cases where periodontitis is present [117].

Alternatively, a causal association of periodontitis with glycemic traits (fasting glucose and insulin, HbA1c) and type 2 diabetes was assessed by mendelian randomization and no genetically instrumented liability to periodontal disease was observed to glycemic traits or diabetes, thus Shah et al. conclude that a causal association of liability to periodontitis with glycemic traits or T2DM is not suported [118]. These results were similar to those reported by Wang that do not endorse a bidirectional causal association between type 2 diabetes and periodontitis [119].

This two-way relationship between diabetes and periodontitis is still not fully elucidated and further clinical and experimental studies are necessary in order to shed light on this matter so that a more effective management of both pathologies can be implemented. 

## 10. Present Possibilities and Future Challenges

Periodontal disease and diabetes are relatively common chronic diseases, with many similarities and etiopathogenic and pathophysiological interrelations. Inflammation, for example, is an essential component in periodontal disease and diabetes, and its contribution to the evolution of both diseases is becoming increasingly documented. 

Diabetes is associated with an increased risk of periodontal disease, as this review argues based on literature data. In the short term, the pocket resolution is independent of metabolic control. In diabetic patients, adjuvant systemic antibiotic administration improves the severity of the pocket but does not provide attachment gain. Without neglecting the importance of metabolic control, prescribing antibiotics is based on the severity of periodontal damage and the inflammatory condition. Metabolic control of diabetes is especially to be considered in the context of oral complications of periodontal disease that appear to be amplified by the pathogenic pathways involved in the micro-vascular complications of diabetes. Reasonable control of diabetes or prediabetes avoids the onset of early periodontal changes, which are less evident to the patient at the beginning of the disease because they do not involve tooth loss.

In turn, the effect of periodontal disease on the control and evolution of diabetes patients appears to be significant. As presented in this review, periodontal disease (as well as obesity and other conditions) can act to initiate or propagate insulin resistance, thus affecting glycemic control. 

Periodontal treatment improves the management of periodontal infection and improves general health, leading to better control of blood glucose in patients with type 2 diabetes. Consequently, given the significant impact of oral complications on quality of life, the prevention of oral pathology and its early management appears to be essential in the care of diabetes.

Even though there a significant effort has been in this area, the therapy of choice of periodontitis is still the removal of supra and subgingival bacterial plaque through mechanical means. This can be aided by other adjuvant therapies such as antibiotics and antiseptic substances, which may or may not be accompanied by the use of adjuvants (local or systemic) such as antiseptics, antimicrobials and host modulators. The improvements obtained through these strategies are temporary and periodontitis can reappear if oral ecology shifts to a pathologic environment once more. Thus, further studies in this field are necessary, with an emphasis on lifestyle modification and discovery of therapies capable of restoring oral eubiosis and preventing dysbiosis (such as prebiotics and probiotics), to deliver significant results in the long-term management of periodontitis and diabetes. 

The common genes imply that periodontal disease is not causally linked to diabetes, but rather that they are both consequences of analogue aberrant inflammatory pathways. Together with genomic sequences variations, epigenetic changes of DNA can modify the genetic blueprint of host response. Thus, future research is needed to assess and extend our knowledge before we can include specific genetic markers in the final cause and effect conclusions. In this regard, ample studies, involving several research centers and subspecialties at the same time, are necessary to materialize the promise of genetic discovery in both pathologies.

## 11. Conclusions 

Although this review has provided up-to-date information on the two diseases and their interrelationships, such interdependent pathogenesis should be further investigated by clinicians (general practitioners, dentists, diabetologists, etc.) and future research studies in order to establish the most appropriate prevention and treatment measures.

Although research in this area is promising, until a more definitive treatment for these diseases is discovered emphasis should be placed on oral health education and the impact that periodontitis can have on the overall inflammatory systemic burden, diabetes evolution and the risk of developing complications.

## Data Availability

Not applicable.
